# Case Report: Diagnosis and management of severe pneumonia caused by complex mixed infection with multidrug-resistant bacteria and fungi after liver retransplantation

**DOI:** 10.3389/fsurg.2026.1903991

**Published:** 2026-07-28

**Authors:** Jiaying Lu, Yingying Xia, Han Wang, Chen Gao, Xingguo Niu, Yibo Zhang, Dalong Zhang

**Affiliations:** The Fifth Clinical Medical College, Henan University of Chinese Medicine, Zhengzhou, Henan, China

**Keywords:** immunosuppression, invasive fungal infection, liver retransplantation, multidrug-resistant organisms, severe pneumonia

## Abstract

**Background:**

Patients undergoing liver retransplantation remain in a prolonged immunosuppressed state and are at high risk for complex mixed infections caused by multidrug-resistant organisms (MDROs) and invasive fungi. Under such circumstances, accurately identifying the predominant pathogens and developing a treatment strategy that balances anti-infective therapy with organ function preservation remain major challenges in current clinical practice.

**Case presentation:**

This article reports the case of a 59-year-old male patient who developed severe pneumonia, septic shock, and multiple organ failure following liver retransplantation. Etiological investigations identified carbapenem-resistant *Klebsiella pneumoniae* (CRKP) harboring NDM+/KPC+, carbapenem-resistant *Acinetobacter baumannii* (CRAB) carrying OXA-23+, along with co-infections caused by *Aspergillus fumigatus*, *Aspergillus flavus*, and *Candida albicans*. Targeted anti-infective therapy was initiated promptly, including aztreonam/avibactam, polymyxins, eravacycline, isavuconazole, and caspofungin during the treatment course. During anti-infective therapy, the patient developed severe bone marrow suppression and gastrointestinal bleeding secondary to coagulation dysfunction. Following tailored modification of the antimicrobial regimen and aggressive supportive management with blood product transfusions, the infection was effectively controlled, and the patient was successfully weaned from mechanical ventilation and discharged after recovery.

**Conclusions:**

For complex mixed infections following liver retransplantation, continuous evaluation of pathogen profiles, immune status, and organ function is critical for guiding individualized treatment strategies. This case highlights that, while potent targeted anti-infective therapy may be life-saving, close monitoring and prompt intervention for treatment-related adverse events and complications are equally essential to achieve a balance between therapeutic benefit and clinical risk under critical conditions.

## 1 Introduction

Liver transplantation is the most effective treatment for end-stage liver disease; however, the prolonged immunosuppressive state following transplantation renders recipients highly susceptible to opportunistic infections. Among these, sepsis and multiple organ failure secondary to pulmonary infection are major causes of mortality after transplantation (1). Furthermore, patients undergoing liver retransplantation face substantially greater challenges, including more complex anatomical reconstruction, more severe ischemia–reperfusion injury, and a higher cumulative burden of immunosuppression. These factors substantially compromise the host immune barrier and exponentially increase the risk of severe postoperative infections (2).

In recent years, the incidence of mixed infections caused by multidrug-resistant organisms (MDROs) and invasive fungi following liver transplantation has increased significantly (3). In particular, recurrent mixed infections involving carbapenem-resistant *Klebsiella pneumoniae* (CRKP) co-producing NDM and KPC carbapenemases, carbapenem-resistant *Acinetobacter baumannii* (CRAB), together with invasive fungal pathogens such as *Aspergillus fumigatus*, are often poorly responsive to conventional anti-infective strategies (4). In these highly drug-resistant infections, therapeutic options are extremely limited, and the use of combined high-grade antimicrobial agents is frequently associated with substantial toxicities, predisposing patients to severe bone marrow suppression and coagulation dysfunction during treatment (5).

This article reports a rare case of severe pulmonary infection following liver retransplantation. The patient developed a critically severe condition characterized by the coexistence of highly drug-resistant bacterial pathogens and invasive fungal infections involving both the airway and systemic circulation. To address this complex clinical scenario, continuous microbiological monitoring and serial reassessment of organ function were performed to guide individualized antimicrobial therapy throughout the clinical course. With the support of mechanical ventilation and multidisciplinary critical care, infection control was achieved, ultimately enabling successful ventilator weaning and clinical recovery. The clinical significance of this case lies in its demonstration of how anti-infective therapy can be adaptively refined in the setting of concurrent extensively drug-resistant bacterial and invasive fungal infections after liver retransplantation, based on evolving etiological evidence, immunosuppressive status, and systemic physiological tolerance, while simultaneously managing severe bone marrow suppression and gastrointestinal bleeding secondary to coagulation dysfunction.

## 2 Case presentation

### 2.1 Patient information and medical history

A 59-year-old male patient was admitted on November 12, 2025, with a chief complaint of “dyspnea for 20 min”. The patient had undergone liver retransplantation and was transferred from the general ward to the intensive care unit (ICU) because of respiratory failure and septic shock. More than 8 months earlier, he had undergone primary allogeneic liver transplantation for hepatocellular carcinoma and hepatitis B virus-related cirrhosis. His postoperative recovery was poor and was complicated by left hepatic lobe necrosis and liver abscess, for which left hepatectomy was performed on May 21, 2025. More than 1 month after surgery, he developed bile leakage and biliary tract infection and subsequently received drainage, anti-infective therapy, and hepatoprotective treatment. On September 21, 2025, he underwent repeat allogeneic liver transplantation combined with adhesiolysis and bile duct exploration with T-tube drainage at our institution.

The patient had a 25-year history of chronic hepatitis B virus infection. After liver transplantation, he received antiviral therapy with tenofovir alafenamide fumarate and immunosuppressive therapy consisting of tacrolimus combined with mycophenolate mofetil for prevention of graft rejection. In December 2024, he was diagnosed with hepatocellular carcinoma complicated by portal vein tumor thrombus and subsequently received four cycles of immunotherapy with atezolizumab plus bevacizumab. He had no history of hypertension or diabetes mellitus and no other prior surgical history apart from procedures related to the present illness.

### 2.2 Clinical presentation and ICU admission

On ICU admission, the patient presented with severe respiratory distress and circulatory instability requiring mechanical ventilation via a tracheostomy tube. His body temperature was 36.7 °C, heart rate was 88 beats/min, and blood pressure was 103/54 mmHg.

Physical examination revealed jaundice of the skin and sclera. The abdomen was distended without tenderness, and a surgical scar approximately 35 cm in length was observed in the upper abdomen. No visible gastric peristalsis or abdominal wall venous dilatation was present. Pulmonary auscultation demonstrated coarse breath sounds bilaterally with audible rhonchi. No pleural friction rub was detected.

Shortly after ICU admission, the patient developed hypotension (85/56 mmHg), accompanied by respiratory failure and decreased oxygen saturation, requiring continued mechanical ventilatory support. Fluid resuscitation and vasoactive therapy with norepinephrine were initiated to maintain hemodynamic stability. The patient also developed anuria, and the Sequential Organ Failure Assessment (SOFA) score was 9, indicating circulatory dysfunction and organ dysfunction secondary to severe infection. Septic shock was therefore suspected.

Given the patient's severe infection and profound immunosuppressed status, prophylactic antimicrobial therapy with valganciclovir and trimethoprim-sulfamethoxazole was not administered upon ICU admission. Valganciclovir carries a risk of bone marrow suppression and accumulation in the presence of renal dysfunction, whereas trimethoprim-sulfamethoxazole may further aggravate cytopenia and is also contraindicated in severe hepatic and renal dysfunction.

The baseline immunosuppressive regimen on ICU admission consisted of tacrolimus (trough concentration, 2.3 ng/mL) combined with mycophenolate mofetil (MMF, 1 g every 12 h). In view of the patient's septic shock and complex mixed infection caused by multidrug-resistant bacteria and invasive fungi, tacrolimus and MMF were discontinued after ICU admission to facilitate recovery of the host immune response. To provide baseline graft protection while addressing the severe stress response associated with sepsis, intravenous methylprednisolone sodium succinate was administered at a dose of 40 mg every 12 h for 7 days.

The patient exhibited manifestations consistent with severe infection and multiple organ dysfunction in the setting of recent liver retransplantation and prolonged immunosuppressive therapy. Based on the clinical presentation, respiratory failure following liver retransplantation complicated by severe pneumonia and septic shock was initially diagnosed.

### 2.3 Initial clinical findings

**Figure 1:**
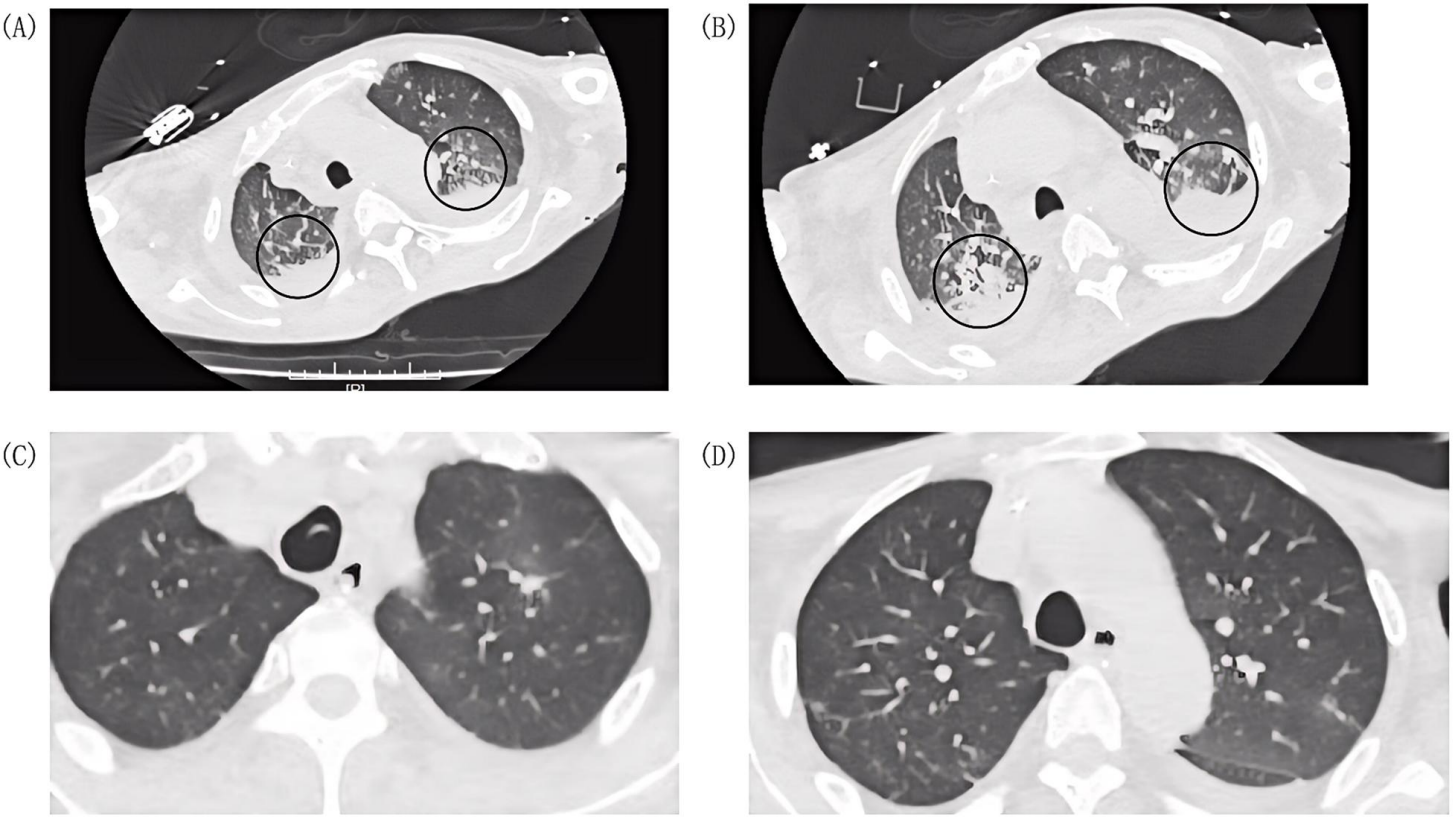
Longitudinal chest CT imaging of the patient. **(A,B)** Initial unenhanced chest CT scans at admission demonstrate extensive inflammatory involvement across bilateral lung fields. The circled areas highlight characteristic features of invasive pulmonary aspergillosis (IPA), including diffuse ground-glass opacities and dense confluent consolidations, predominantly distributed in the peripheral zones of the lower lobes. **(C,D)** Follow-up chest CT scans after targeted antimicrobial and antifungal therapy show near-complete resolution of the previous lesions. The lung parenchyma is well-aerated with no residual focal opacities, indicating a substantial radiological improvement and a favorable clinical response.

Chest computed tomography (CT) demonstrated extensive bilateral pulmonary inflammatory infiltrates (Figures 1A,B). Respiratory mechanics monitoring revealed increased airway resistance and reduced lung compliance, suggesting severe airway obstruction and pulmonary parenchymal involvement.

Given the patient's immunocompromised status and persistent respiratory deterioration, repeated bedside fiberoptic bronchoscopy was performed after ICU admission. Bronchoscopic examination revealed a large amount of thick yellow-white mucus plugs within the airway, accompanied by mucosal congestion, edema, and focal mucosal bleeding. Bronchoalveolar lavage fluid (BALF) specimens were collected for microbiological investigation, providing important evidence for subsequent diagnostic evaluation and targeted antimicrobial therapy.

## 3 Timeline

The major clinical events, diagnostic assessments, antimicrobial interventions, complications, and outcomes during hospitalization are summarized in Table 1.

**Table 1:** Timeline of the patient's clinical course.

Time point	Clinical status	Microbiological and laboratory findings	Therapeutic interventions	Rationale for therapeutic modifications
Day 0 (Nov 12–13, 2025) Empirical treatment for sepsis	Septic shock and respiratory failure requiring mechanical ventilation and vasopressor support	Markedly decreased WBC and platelet counts; markedly elevated PCT and CRP	Initiated: imipenem-cilastatin, teicoplanin, colistin, caspofungin	Empirical broad-spectrum coverage for Gram-positive pathogens (including MRSA and *Enterococcus* spp.), Gram-negative pathogens (including CRKP and CRAB), and fungi based on liver retransplantation, prolonged immunosuppression, tracheostomy, and septic shock.
Day 3 Definitive pathogen identification; targeted coverage for CRKP, CRAB, and *Aspergillus* spp.	Sedated; respiratory and circulatory support maintained	BALF mNGS/culture identified *Aspergillus fumigatus*, CRKP, CRAB, and *Enterococcus faecium*. Chest and abdominal CT demonstrated bilateral pulmonary infection and intra-abdominal abscesses.	Added: aztreonam-avibactam, isavuconazonium sulfate Continued: colistin, teicoplanin Discontinued: imipenem-cilastatin, caspofungin	mNGS confirmed NDM/KPC-producing CRKP, supporting aztreonam/avibactamtherapy. Caspofungin was replaced with isavuconazonium sulfate because of suspected *Aspergillus* infection and its more favorable hepatic safety profile. imipenem-cilastatin was discontinued because of CRKP resistance.
Day 7 Intensified antifungal therapy	Respiratory status relatively stable; persistent circulatory instability	Chest and abdominal CT showed bilateral pulmonary infection, pulmonary edema, pleural effusion, encapsulated intra-abdominal abscesses, and newly developed pericardial effusion.	Added: nebulized amphotericin B Continued: isavuconazonium sulfate, aztreonam-avibactam, colistin, teicoplanin	Imaging findings and serological evidence remained suggestive of invasive aspergillosis. Nebulized amphotericin B was added to increase local pulmonary drug exposure, while antibacterial therapy was maintained.
Day 14 Airway infection control and improvement of respiratory mechanics	Failed ventilator weaning; uncontrolled infection; copious tenacious airway secretions	Repeated critical thrombocytopenia (6–12 × 10⁹/L), marked coagulopathy, and positive fecal occult blood test	Added: eravacycline Continued: aztreonam-avibactam, colistin, isavuconazonium sulfate, nebulized amphotericin B Discontinued: teicoplanin Supportive therapy: repeated platelet, plasma, and cryoprecipitate transfusions	Eravacycline was added because of inadequate control of CRAB-associated intra-abdominal infection. Blood component transfusion was initiated because of worsening bleeding risk.
Day 18 Optimization of antifungal therapy; correction of coagulopathy and severe anemia	Ventilator-dependent; severe anemia and worsening coagulopathy	Persistent critical thrombocytopenia, progressive decline in hemoglobin, and marked prolongation of PT and APTT	Reinitiated: caspofungin Continued: eravacycline, colistin, isavuconazonium sulfate, nebulized amphotericin B Supportive therapy: repeated platelet, plasma, and cryoprecipitate transfusions	Progressive imaging findings suggested severe invasive aspergillosis. Triple antifungal therapy with isavuconazonium sulfate, caspofungin, and nebulized amphotericin B was implemented.
Day 35 (Dec 7, 2025) Clinical improvement and bleeding control	Successfully weaned from mechanical ventilation; gastrointestinal bleeding controlled	Hemoglobin 52 g/L; platelet count 6 × 10⁹/L	Continued: antimicrobial therapy (adjusted according to antimicrobial susceptibility testing); vasopressors discontinued Supportive therapy: CRRT, hepatoprotective therapy, nutritional support, and massive blood component transfusion	Gastrointestinal bleeding was controlled, mechanical ventilation was successfully discontinued, the patient was transferred to the general ward, and antimicrobial therapy was subsequently de-escalated.

## 4 Diagnostic assessment, therapeutic intervention, follow-up and outcomes

### 4.1 Diagnostic assessment

To evaluate airway involvement and identify the causative pathogens, the patient underwent repeated bedside fiberoptic bronchoscopy. Bronchoscopic examination revealed abundant yellow-white tenacious mucus plugs accompanied by mucosal congestion, edema, and focal mucosal hemorrhage. During the procedure, bronchoalveolar lavage fluid (BALF) samples were collected simultaneously for metagenomic next-generation sequencing (mNGS) and conventional microbiological culture. The results demonstrated a complex mixed lower respiratory tract infection caused by carbapenem-resistant *Klebsiella pneumoniae* (CRKP), carbapenem-resistant *Acinetobacter baumannii* (CRAB), *Aspergillus fumigatus*, *Aspergillus flavus*, and *Candida albicans*.

Further analysis of the resistance mechanisms demonstrated that the CRKP isolate co-produced NDM and KPC carbapenemases, whereas the CRAB isolate harbored the OXA-23 carbapenemase gene. This extensive resistance profile left very limited options for targeted antimicrobial therapy.

Regarding the fungal infection, the patient was considered to have probable invasive pulmonary aspergillosis (IPA) rather than simple airway colonization. Airway colonization is typically characterized by isolation of *Aspergillus* species from respiratory specimens without evidence of tissue invasion or compatible radiological progression. In contrast, this patient fulfilled a comprehensive diagnostic framework for IPA. First, BALF analysis identified *Aspergillus fumigatus* and other fungal pathogens. Second, the initial serum galactomannan (GM) index on admission was 1.108 S/CO, followed by a progressive decline to 0.577 S/CO and subsequently to <0.100 S/CO after antifungal therapy, meeting the mycological criterion defined by the EORTC/MSGERC consensus (serum GM index ≥1.0). In addition, chest computed tomography demonstrated bilateral patchy consolidations with partial cavitary lesions, which were highly compatible with the characteristic radiological features of IPA. Taken together with the patient's profoundly immunocompromised status, compatible imaging findings, positive mycological evidence, and elevated serum GM level, these findings strongly supported a diagnosis of probable IPA rather than airway colonization.

The microbiological characteristics are summarized in Table 2.

**Table 2:** Microbiological identification profile.

No.	Pathogens	Specimen source/Diagnostic method	MIC (minimum inhibitory concentration)	Timing of culture sampling for different specimens	Quantitative mNGS results	Sequencing reads	Blood culture findings	Galactomannan (GM) levels	(1,3)-β-D-glucan (BDG) levels
1	*Klebsiella pneumoniae* (CRKP)	BALF; mNGS and Culture	Imipenem >8 (R); Tetracycline >8 (R); Aztreonam >32 (R); Tigecycline 2 (S); Moxifloxacin >2 (R); Colistin ≤1 (S)	2025/11/14	Carbapenemase genes (NDM, KPC)	11,256	On November 12, 2025, two sets of blood cultures were negative; a rapid bloodstream infection 13-item panel detected Candida spp. and *Enterococcus* spp.		
2	*Acinetobacter baumannii* (CRAB)	BALF; mNGS and Culture	Imipenem >8 (R); Minocycline ≤1 (S); Amikacin ≤8 (S); Cefoperazone-sulbactam >32/8 (R); Tigecycline 2 (S); Colistin ≤1 (S)	2025/11/12	*Acinetobacter baumannii* complex	9,782	Two sets of blood cultures were negative; however, a rapid 13-item bloodstream infection panel detected *Candida* spp. and *Enterococcus* spp.		
3	*Aspergillus fumigatus/A. flavus*	BALF; mNGS and Culture		2025/11/14	*Aspergillus fumigatus/Aspergillus flavus*	*Aspergillus flavus* (36 reads); *Aspergillus fumigatus* (23 reads)	Two sets of blood cultures and fungal blood cultures were negative	2.659 S/CO (elevated)	568.318 pg/mL (Markedly elevated)
4	*Candida albicans*	BALF; mNGS and Culture; Serial monitoring of serum (1,3)-β-D-glucan (BDG)		2025/11/14	*Candida* spp.	*Candida albicans* (58 reads)	Two sets of blood cultures and fungal blood cultures were negative		

BALF, bronchoalveolar lavage fluid; mNGS, metagenomic next-generation sequencing; IV, intravenous; qd, once daily; q12h, every 12 h; q8h, every 8 h.

### 4.2 Therapeutic intervention

The patient was diagnosed with a complex mixed infection involving NDM- and KPC-producing carbapenem-resistant *Klebsiella pneumoniae* (CRKP), multidrug-resistant *Acinetobacter baumannii* (CRAB), and invasive fungal pathogens, including *Aspergillus fumigatus*, *Aspergillus flavus*, and *Candida albicans*. Given the patient's profound immunosuppression and multiorgan dysfunction following liver transplantation, the antimicrobial regimen was continuously optimized throughout the clinical course based on microbiological findings, antimicrobial availability, and the patient's physiological reserves.

Before bronchoalveolar lavage (BAL) microbiological results and NDM/KPC resistance profiles became available, the patient required immediate empiric broad-spectrum antimicrobial therapy because of septic shock. Imipenem was therefore administered during the initial phase of treatment. Once BAL specimens confirmed NDM- and KPC-producing carbapenem-resistant *Klebsiella pneumoniae* (CRKP), the antimicrobial regimen was switched to aztreonam combined with avibactam as the core targeted therapy, taking into account the limited availability of ceftazidime/avibactam at that time. This regimen was selected because it provides complementary activity against both carbapenemase mechanisms: aztreonam remains stable in the presence of metallo-β-lactamases such as NDM, whereas avibactam effectively inhibits serine β-lactamases, including KPC, thereby extending antimicrobial activity against both resistance mechanisms. Drug preparation and administration were performed according to standardized institutional protocols. Considering the patient's renal insufficiency, the dosage was adjusted and the dosing interval was extended to optimize drug exposure while minimizing the risk of drug accumulation and nephrotoxicity.

For recurrent isolation of carbapenem-resistant *Acinetobacter baumannii* (CRAB), the baseline regimen consisted of intravenous polymyxin E combined with adjunctive inhaled administration to achieve adequate systemic drug exposure while increasing antimicrobial concentrations within the lower respiratory tract. Cefiderocol and sulbactam-containing regimens were not selected, and eravacycline was added to enhance anti-CRAB coverage based on three clinical considerations. First, the patient had concurrent pulmonary and intra-abdominal CRAB infections, and eravacycline has demonstrated favorable penetration into both pulmonary and intra-abdominal tissues. Second, given the patient's renal impairment following liver transplantation, eravacycline was selected because of its favorable hepatic and renal tolerability, whereas cefiderocol and high-dose sulbactam-containing regimens may result in greater renal drug exposure. Third, antimicrobial susceptibility testing showed that the isolated CRAB strain exhibited greater *in vitro* susceptibility to eravacycline than to cefiderocol or ampicillin/sulbactam. Taken together, these findings supported the addition of eravacycline to the polymyxin E–based regimen to optimize antimicrobial coverage against this extensively drug-resistant isolate.

Given the patient's prolonged immunosuppressed state, bronchoalveolar lavage (BAL) identified a mixed fungal infection involving *Aspergillus fumigatus*, *Aspergillus flavus*, and *Candida albicans*. Systemic antifungal therapy consisted of isavuconazole in combination with caspofungin, with adjunctive inhaled amphotericin B administered to increase local antifungal drug exposure within the lower respiratory tract. Isavuconazole was selected because of its broad activity against *Aspergillus* species and its favorable hepatic safety profile, making it particularly suitable for patients following liver transplantation. Caspofungin was added to provide additional coverage against *Candida* species, while inhaled amphotericin B was used to further increase local drug exposure within the airways. This multimodal antifungal strategy was implemented in response to persistent pulmonary cavitary lesions and continuously elevated fungal biomarkers, with the goal of improving local infection control while maintaining systemic antifungal efficacy.

In addition to pathogen-directed antimicrobial therapy, comprehensive multiorgan supportive care was implemented throughout the patient's clinical course. During septic shock, continuous hemodynamic monitoring was performed, and vasoactive agents were administered to maintain circulatory stability. Respiratory support included invasive mechanical ventilation, airway clearance, pulmonary rehabilitation, and repeated bronchoscopy with bronchoalveolar lavage (BAL) for airway management and microbiological reassessment. Post-transplant management consisted of hepatoprotective therapy, stress ulcer prophylaxis, routine surveillance of graft perfusion, and early enteral nutrition via a nasojejunal tube. Standardized sedation and analgesia protocols were combined with bedside rehabilitation to preserve neuromuscular function. In addition, infection-related bone marrow suppression and coagulopathy were managed with transfusion of packed red blood cells, platelets, fresh frozen plasma, and cryoprecipitate as clinically indicated to correct anemia, restore coagulation function, and control bleeding.

### 4.3 Follow-up and outcomes

Following antimicrobial optimization and comprehensive supportive care, inflammatory markers gradually declined and respiratory function progressively improved. Respiratory mechanics monitoring demonstrated a reduction in airway resistance and improvement in lung compliance. The patient was successfully liberated from mechanical ventilation after approximately 5 weeks of treatment.

Follow-up chest computed tomography demonstrated marked resolution of bilateral pulmonary infiltrates and inflammatory lesions compared with baseline imaging (Figures 1C,D).

On December 7, 2025, the patient developed acute gastrointestinal hemorrhage, with a hemoglobin level of 52 g/L and a platelet count of 6 × 10⁹/L. Emergency hemostatic therapy and blood product transfusions were promptly administered, resulting in stabilization of the patient's condition.

The gastrointestinal bleeding was considered multifactorial and may have been associated with severe coagulopathy, liver dysfunction, anticoagulation exposure, and prolonged treatment with multiple antimicrobial agents. Following intensive supportive management, the patient recovered steadily, was transferred to a general ward, and was subsequently discharged in stable condition.

## 5 Discussion

### 5.1 Mechanisms of infection following liver retransplantation

Compared with primary liver transplantation, liver retransplantation is associated with a substantially higher risk of severe infection because of cumulative immunosuppressive exposure, repeated surgical trauma, and disruption of normal anatomical barriers (2, 6). In the present case, prolonged immunosuppressive therapy with tacrolimus and mycophenolate mofetil, together with pancytopenia and splenic infarction, resulted in profound impairment of host immune defenses and increased susceptibility to opportunistic pathogens (6, 9, 20).

Furthermore, repeated major abdominal procedures, including partial hepatectomy, biliary drainage, two liver transplantations, and adhesiolysis, caused tissue injury, local ischemia, and impairment of natural barrier function. Prolonged use of invasive devices, including tracheostomy tubes, central venous catheters, and enteral feeding tubes, further increased the risk of healthcare-associated infections and multidrug-resistant organism colonization (7, 10). A recent large single-center retrospective study with a 13-year follow-up also demonstrated the high incidence of postoperative infections and the substantial challenges associated with infection management in liver transplant recipients (22). The coexistence of these factors ultimately contributed to the development of complex mixed infections involving multidrug-resistant bacteria and invasive fungi (3, 5, 8).

### 5.2 Longitudinal microbiological surveillance in complex mixed infections

The successful management of this case relied heavily on continuous microbiological monitoring and timely optimization of antimicrobial therapy. The identification of NDM, KPC, and OXA-23 resistance determinants by mNGS, together with conventional culture results, enabled early recognition of extensive antimicrobial resistance and facilitated pathogen-directed treatment (15).

As summarized in Table 3, serial evaluation of microbiological findings, inflammatory biomarkers, and clinical response guided antimicrobial escalation and de-escalation throughout the treatment course. This approach minimized unnecessary antimicrobial exposure while maintaining adequate pathogen coverage and reducing the risk of treatment-related toxicity (23).

**Table 3:** Dynamic microbiological monitoring and targeted antimicrobial therapy strategies for complex mixed infections.

Target pathogen	Monitoring method	Therapeutic adjustment	Pharmacological and clinical rationale
Dual-carbapenemase-producing CRKP	BALF-mNGS identified both KPC and NDM genes, indicating the coexistence of a serine carbapenemase and a metallo-β-lactamase.	Aztreonam/avibactam	Avibactam inhibits KPC enzymes, thereby protecting aztreonam from hydrolysis. Because aztreonam remains stable against NDM-mediated hydrolysis, the combination provides comprehensive coverage against both resistance mechanisms (11).
CRAB	Confirmed by sputum and ascitic fluid cultures; mNGS detected the OXA-23 gene, and antimicrobial susceptibility testing demonstrated carbapenem resistance.	Polymyxin E (intravenous plus inhaled) combined with eravacycline	Eravacycline enhances ribosomal binding affinity and retains activity against strains with efflux pump-mediated resistance. Inhaled polymyxin E was administered to increase drug exposure at the alveolar target site (12).
Invasive aspergillosis	BALF-mNGS detected *Aspergillus fumigatus* sequences; serum galactomannan (GM) levels remained elevated, and chest imaging revealed pulmonary nodules.	Isavuconazole replaced caspofungin and was combined with inhaled amphotericin B.	Isavuconazole offers a broader antifungal spectrum and exhibits fewer cytochrome P450-mediated drug interactions, resulting in less interference with graft function and immunosuppressive therapy. Adjunctive inhaled amphotericin B may improve local pulmonary drug exposure where systemic penetration is limited (13, 14).
Complex mixed infection	Dynamic monitoring of inflammatory biomarkers (PCT and CRP) and pathogen burden (mNGS sequence counts).	Antimicrobial therapy was escalated or de-escalated according to microbiological findings and clinical response.	This strategy minimizes the risk of secondary infections and microbiota disruption associated with prolonged broad-spectrum antimicrobial exposure while maintaining adequate pathogen coverage and balancing treatment-related toxicities, including bone marrow suppression and coagulation abnormalities, against therapeutic benefit (5).

Compared with previously reported infections after liver transplantation, this case was unusual because of the simultaneous coexistence of dual-carbapenemase-producing CRKP, OXA-23-positive CRAB, and multiple invasive fungal pathogens (3–5, 9). Reports describing this pathogen combination following liver retransplantation remain limited, highlighting the clinical challenges associated with diagnosis and treatment in this highly vulnerable population (4, 9).

### 5.3 Respiratory mechanics and ventilator weaning after infection control

Severe airway obstruction caused by inflammatory secretions, mucus plugging, and diffuse pulmonary inflammation was the principal barrier to ventilator liberation in this patient. Bronchoscopic examination repeatedly demonstrated extensive airway secretions and mucosal inflammation, which contributed to increased airway resistance and impaired lung compliance.

Repeated bronchoscopy combined with bronchoalveolar lavage not only facilitated airway clearance but also provided valuable microbiological specimens for pathogen identification (15, 16). Following optimization of antimicrobial therapy, inflammatory markers gradually decreased, respiratory mechanics improved, and chest imaging demonstrated progressive resolution of pulmonary infiltrates (17).

Successful ventilator weaning was therefore closely associated with effective infection control. In addition to microbiological evidence and radiological improvement, serial assessment of respiratory mechanics provided useful information regarding pulmonary recovery and readiness for liberation from mechanical ventilation (17, 18).

### 5.4 Therapeutic benefits and treatment-related complications

Although aggressive antimicrobial therapy was necessary to control life-threatening multidrug-resistant bacterial and fungal infections, treatment was accompanied by substantial clinical challenges (5). The patient developed severe bone marrow suppression, coagulopathy, and gastrointestinal hemorrhage during the treatment course. Following hematology consultation, bone marrow examination was recommended to further evaluate the cause of the hematologic abnormalities. However, the patient's family declined the procedure. Therefore, the following discussion is based on clinical inference, and the possibility that these hematologic abnormalities were secondary to severe infection cannot be excluded.

Multiple factors likely contributed to these complications. Long-term immunosuppressive therapy, particularly mycophenolate mofetil, may have impaired hematopoiesis and reduced platelet production (19). Persistent systemic inflammation, repeated surgical interventions, hepatic dysfunction, and prolonged critical illness likely further aggravated coagulation abnormalities and bleeding risk (21). In addition, cumulative toxicities associated with multiple antimicrobial agents may have contributed to hematologic and organ-related adverse effects (5, 23).

This case emphasizes that successful management of complex infections after liver retransplantation requires more than effective pathogen eradication alone. Continuous reassessment of antimicrobial efficacy, drug-related toxicity, coagulation status, and end-organ viability is essential to achieve an individualized balance between therapeutic benefit and clinical risk.

### 5.5 Limitations and lessons learned

Several limitations should be acknowledged. As a single-case report, the findings cannot be generalized to all liver retransplant recipients with severe mixed infections. In addition, the relative contribution of individual antimicrobial agents to clinical recovery could not be precisely determined because multiple therapies were administered simultaneously.

Despite these limitations, several important lessons can be drawn from this case. First, early pathogen identification using mNGS combined with conventional microbiological testing can facilitate timely implementation of targeted therapy in complex mixed infections. Second, antimicrobial regimens should be promptly tailored according to evolving microbiological findings, immune status, and organ function. Finally, close monitoring and prompt management of treatment-related complications are equally important as infection control in critically ill transplant recipients.

Overall, this case highlights the importance of integrating microbiological evidence, immune status, and organ reserve evaluation when managing severe multidrug-resistant bacterial and fungal infections following liver retransplantation. Such an individualized approach may improve the likelihood of successful infection control while minimizing treatment-related complications.

## 6 Patient perspective (from the perspective of the patient's family)

### 6.1 Patient perspective

As the patient's family, witnessing his sudden respiratory failure and admission to the intensive care unit after liver retransplantation was an extremely stressful experience. We were informed of the severity of the mixed infection caused by multidrug-resistant bacteria and invasive fungi, as well as the potential toxicities of the intensive antimicrobial therapy, which helped us understand the complexity of his condition and treatment. During the five weeks of mechanical ventilation, his clinical condition fluctuated repeatedly, and the episode of massive gastrointestinal bleeding following successful ventilator weaning was particularly distressing. Throughout his hospitalization, the medical team maintained clear and timely communication with our family, explained each treatment decision, and adjusted the management plan according to his changing condition. We greatly appreciated the multidisciplinary care provided by the intensive care and transplant teams, and we are grateful that he ultimately recovered sufficiently to be discharged home.

## Data Availability

The original contributions presented in the study are included in the article/Supplementary Material, further inquiries can be directed to the corresponding author.

## References

[1] Qian YB Chen F Hang HL Shen C Han LZ Deng YX. Risk factors and outcomes of early infection in liver transplant recipients with acute-on-chronic liver failure. J Dig Dis. (2022) 23(11):642–50. 10.1111/1751-2980.13151 36617995

[2] Dakroub A Anouti A Cotter TG Lee WM. Mortality and morbidity among adult liver retransplant recipients. Dig Dis Sci. (2023) 68(10):4039–49. 10.1007/s10620-023-08065-2 37597085

[3] Martin-Mateos R Martínez-Arenas L Carvalho-Gomes Á Aceituno L Cadahía V Salcedo M. Multidrug-resistant bacterial infections after liver transplantation: prevalence, impact, and risk factors. J Hepatol. (2024) 80(6):904–12. 10.1016/j.jhep.2024.02.023 38428641

[4] Collis B Urbancic K Whitelaw J Reynolds G Vogrin S Jahanabadi H. Outcomes of invasive aspergillosis in liver transplant recipients from an institution using targeted antifungal prophylaxis and an antifungal stewardship program. Transpl Infect Dis. (2025) 27(4):e70046. 10.1111/tid.70046 40356260

[5] Ferrarese A Senzolo M Sasset L Bassi D Cillo U Burra P. Multidrug-resistant bacterial infections in the liver transplant setting. Updates Surg. (2024) 76(7):2521–9. 10.1007/s13304-024-01903-6 38918314 PMC11602820

[6] Peters AL Tremblay S Alloway RR Woodle ES. Immunosuppression for liver retransplantation: Babel revisited. Transplantation. (2021) 105(8):1658–9. 10.1097/TP.0000000000003418 32804804

[7] Shinde AS Kapoor D. Infections after liver transplant -timeline, management and prevention. J Clin Exp Hepatol. (2024) 14(3):101316. 10.1016/j.jceh.2023.101316 38264574 PMC10801311

[8] Hao Q Wang H Zhang Q Guo F Liu X Zhang L. Risk factors for multidrug-resistant bacterial infections after liver transplantation. Ann Transplant. (2025) 30:e949047. 10.12659/AOT.949047 41054187 PMC12514944

[9] Karakaş EE Toplu SA Şahin TT Karakaş S Satılmış B Akbulut S. Infections in liver retransplantation: a prospective study of microbial patterns, immune biomarkers, and survival. Transplant Proc. (2026) 58(3):532–9. 10.1016/j.transproceed.2026.02.004 41763944

[10] Schreiber PW Hoessly LD Boggian K Neofytos D Van Delden C Egli A. Surgical site infections, risk factors, and outcomes after liver transplant. JAMA Netw Open. (2025) 8(3):e251333. 10.1001/jamanetworkopen.2025.1333 40116828 PMC11929024

[11] Qi G Cong W Zhang M Wang S Bai Z Li B. Combination therapy with ceftazidime-avibactam, aztreonam, and topical polymyxin B for NDM-producing *Klebsiella pneumoniae* CNS infection: a case report. Infect Drug Resist. (2026) 19:608393. 10.2147/IDR.S608393 42152881 PMC13180328

[12] Mimram L Timsit JF Rondinaud E Le M Thy M. Eravacycline as a last resort for difficult-to-treat resistant *Acinetobacter baumannii* infections in critically ill patients: three case reports with pharmacokinetic insights. JAC Antimicrob Resist. (2025) 7(3):dlaf095. 10.1093/jacamr/dlaf095 40583997 PMC12203001

[13] Zhang T Shen Y Feng S. Clinical research advances of isavuconazole in the treatment of invasive fungal diseases. Front Cell Infect Microbiol. (2022) 12:1049959. 10.3389/fcimb.2022.1049959 36530445 PMC9751058

[14] Caballero-Bermejo AF Darnaude-Ximénez I Aguilar-Pérez M Gomez-Lopez A Sancho-López A López García-Gallo C. Bronchopulmonary penetration of isavuconazole in lung transplant recipients. Antimicrob Agents Chemother. (2023) 67(10):e0061323. 10.1128/aac.00613-23 37787528 PMC10583689

[15] Gu W Miller S Chiu CY. Clinical metagenomic next-generation sequencing for pathogen detection. Annu Rev Pathol. (2019) 14:319–38. 10.1146/annurev-pathmechdis-012418-012751 30355154 PMC6345613

[16] Benavente K Yamasaki K Daoud E. Flexible bronchoscopy during mechanical ventilation. Why and why not. J Mech Vent. (2022) 3:34–40. 10.53097/JMV.10044

[17] Gertler R. Respiratory mechanics. Anesthesiol Clin. (2021) 39(3):415–40. 10.1016/j.anclin.2021.04.003 34392877 PMC8360707

[18] Geiseler J Westhoff M. Weaning von invasiver beatmung [weaning from invasive mechanical ventilation]. Med Klin Intensivmed Notfmed. (2021) 116(8):715–26. 10.1007/s00063-021-00858-5 34586430 PMC8479264

[19] Staatz CE Tett SE. Clinical pharmacokinetics and pharmacodynamics of mycophenolate in solid organ transplant recipients. Clin Pharmacokinet. (2007) 46(1):13–58. 10.2165/00003088-200746010-00002 17201457

[20] Jain A Reyes J Kashyap R Rohal S Abu-Elmagd K Starzl T. What have we learned about primary liver transplantation under tacrolimus immunosuppression? Long-term follow-up of the first 1000 patients. Ann Surg. (1999) 230(3):441–9. 10.1097/00000658-199909000-00016 10493490 PMC1420888

[21] Lisman T Porte RJ. Rebalanced hemostasis in patients with liver disease: evidence and clinical consequences. Blood. (2010) 116(6):878–85. 10.1182/blood-2010-02-261891 20400681

[22] Kurt EK Bulut R Kandemir B Keskin PB Şentürk M Bıyık M. Evaluation of infections developing in liver transplant patients: thirteen-year experience of a single center. Transplant Proc. (2026) 58:760–8. 10.1016/j.transproceed.2026.03.013 41997770

[23] Timsit J-F Bassetti M Cremer O Daikos G De Waele J Kallil A. Rationalizing antimicrobial therapy in the ICU: a narrative review. Intensive Care Med. (2019) 45(2):172–89. 10.1007/s00134-019-05520-5 30659311

